# Rapid testing of red blood cell parameters in primary care patients using HemoScreen™ point of care instrument

**DOI:** 10.1186/s12875-019-0971-2

**Published:** 2019-06-07

**Authors:** Anders Larsson, Lena Carlsson, Bo Karlsson, Miklos Lipcsey

**Affiliations:** 1 Department of Medical Sciences, Section Clinical Chemistry, Uppsala University, Akademiska sjukhuset, entrance 61, 3rd floor, SE-751 85 Uppsala, Sweden; 2 Department of Public Health and Caring Sciences, Uppsala University, Uppsala, Sweden; 3 The Hedenstierna Laboratory, CIRRUS, Department of Surgical Sciences, Anaesthesiology and Intensive Care Medicine, Uppsala University, Uppsala, Sweden

**Keywords:** Anemia, Iron deficiency, red blood cells, Method evaluation, Point of care testing, Primary care

## Abstract

**Background:**

Patients with anemia are frequently encountered in primary care. Once anemia is detected, it is essential to define the type and identify the underlying cause prior to initiation of treatment. In most cases, the cause can be determined using information from the patient history, physical exam, and complete blood counts (CBC). Point of care testing of blood cell counts would speed up the work up of anemia patients. The aim of the present study was to evaluate if the HemoScreen™ instrument (PixCell Medical, Yokneam Ilit, Israel) could be used for primary care samples. It is a POCT instrument that utilizes single sample cuvettes and image analysis of full blood count including RBC, Hemoglobin, MCV, MCH, platelets, WBC, and WBC 5-part differential.

**Methods:**

We compared the HemoScreen™ and the Sysmex XN instrument results of 100 primary care patient samples focusing on the total white blood cells, red blood cell parameters RBC, Hemoglobin, MCH, MCV and platelets.

**Results:**

Deming correlations between the HemoScreen™ and the Sysmex XN instruments for the CBC were WBC_HemoScreen™_ = 1.016* WBC_Sysmex_ + 0.34; r = 0.981, RBC_HemoScreen™_ = 0.988* RBC_Sysmex_ + 0.015; r = 0.974, Hemoglobin_HemoScreen™_ = 1.081* Hemoglobin_Sysmex_ − 11.25; r = 0.964, MCH_HemoScreen™_ = 0.978* MCH_Sysmex_ + 0.78; r = 0.939, MCV_HemoScreen™_ = 0.963* MCV_Sysmex_ + 8.68; r = 0.946, Platelets_HemoScreen™_ = 0.964* Platelets_Sysmex_ + 25.7; r = 0.953.

**Conclusion:**

The HemoScreen™ instrument could provide rapid and accurate test results for evaluation of the red blood cell parameters in primary care. This new technology is interesting as it allows the analysis red blood cell parameters also at small primary care centers.

## Background

Anemia is one of our major health problems and the World Health Organization estimates that approximately 30% of the population in the world suffers from anemia [1]. The most common cause of anemia is iron deficiency [2, 3]. Anemia patients are often first encountered and often handled in primary care [4] but the signs and symptoms of anemia are nonspecific and may be difficult to detect [5, 6]. It is thus important to use laboratory markers for the detection of anemia and to determine the severity [7]. Even if iron deficiency is the most frequent cause of anemia, it is important to define the cause of the anemia to ensure a rapid and efficient treatment [8]. Other causes of anemia are for instance acute or chronic bleeding, cobalamin or folate deficiencies, hemolytic anemias, malignancies, chronic inflammation, reduced synthesis of red blood cells in the bone marrow, reduced erythropoietin production and hemoglobinopathies [4]. Hemoglobin, mean corpuscular volume (MCV) and mean corpuscular hemoglobin (MCH) are the parameters usually recommended as the first line of investigation of suspected anemia in primary care [9, 10]. The normal range for MCV is from 82 to 98 fL [11]. Elevated levels are seen in patients with vitamin B12 or folic acid deficiency while low values indicate a microcytic anemia [12]. Low MCH values are typically seen in iron deficiency and thalassemia, while increased values occur in macrocytosis [13]. The addition of MCV and MCH thus add valuable information to the hemoglobin value [9]. In Uppsala county there are approximately 160 CBC and 220 hemoglobin test requests from primary care facilities per 1000 inhabitants [8]. Today, all primary care centers in Uppsala County have the ability to perform point-of-care (POC) testing for hemoglobin and the larger primary care centers can analyze MCV and MCH using small cell counters.

This study presents a new technology for cell counting and identification. The gold standard method for identification of cells are manual microscopy. Cells are better differentiated based on their morphology and staining characteristics than in the automated analyzers. Manual microscopy is a time consuming methodology that requires highly trained staff. Manual microscopy has thus often been replaced by cell counters. In the present study we used a Sysmex XN instrument as comparison. The Sysmex XN is a modern cell counter. Cell counters can perform the testing rapidly but a major drawback with cell counters is that only the total fluorescent/scattered light emitted by the cell is measured, and no subcellular data. The cell counters require lasers and complex optics which make them expensive. They also use hydrodynamic focusing that requires large amounts of sheath fluid which causes a burden on reagent handling. The actual flow chamber is small and susceptible to clogging requiring careful maintenance and toxic reagents. The instrument cost and the need for continuous maintenance makes it difficult to use cell counters in decentralized settings.

Imaging cytometry such as the CellaVision DM96 are automated microscopy systems that identifies cells based on the morphology and staining characteristics. They are often used in the centralized laboratories complementing the cell counters when it is suspected they are unable to correctly identify the cells. These are however even more complex, require experienced staff and most importantly do not provide absolute counts.

PixCell Medical has recently developed the HemoScreen™, a POC hematology analyzer, which combines flow cytometry and digital imaging in a single platform [14]. The HemoScreen employs a novel method called viscoelastic focusing which is superior to the traditional hydrodynamic focusing as it does not require sheath fluid. The sharp focusing effect allows the usage of high numerical aperture optics and eliminating the need for sophisticated mechanics. The HemoScreen is designed to operate at point-of-care (POC) settings by its simple usage and minimal maintenance. Once the cartridge is inserted into the analyser the preparation of the sample and its analysis are performed automatically.

The HemoScreen™ requires a sample of 40 μL for the measurement and employs a disposable cartridge, which contains all required reagents. The blood is introduced into the single use cartridge, it is then inserted into the analyzer and the results are displayed within 6 min. The simplicity of the instrument allows the use of the HemoScreen instrument in decentralized settings, e.g. at primary care units that are not staffed with laboratory technicians.

The parameters analyzed by the HemoScreen™ are red blood cells, white blood cells, platelets, hemoglobin, hematocrit, mean corpuscular volume, mean cell hemoglobin, mean cell hemoglobin concentration, red blood cell distribution width, mean platelet volume, neutrophil granulocytes (number and percentage), monocytes (number and percentage), lymphocytes (number and percentage), eosinophils (number and percentage) and basophils (number and percentage) [14].

The aim of this study was to evaluate the performance of the HemoScreen™ instrument for the workup of patients with suspected anemia with the focus on red blood cells (RBC), hemoglobin (HGB), hematocrit (HCT), mean corpuscular volume (MCV), mean cell hemoglobin (MCH) and mean cell hemoglobin concentration (MCHC).

## Methods

### Study population

The samples used were routine requests from primary care physicians in the county of Uppsala and sent to the Department of Clinical Chemistry and Pharmacology, Uppsala University Hospital, Uppsala. The K_2_-EDTA tubes (BD Vacutainer tube 354,664, Becton Dickinson, Franklin Lakes, NJ, USA) were first analyzed on the Sysmex XN instrument and then analyzed with the HemoScreen™ instrument (PixCell Medical, Yokneam Ilit, Israel).

The Uppsala University ethical committee approved the method comparison study (01–367). The ethical permit limits the patient information to age and sex and the samples had to be surplus samples without patient identity. As the samples were surplus samples without any patient identity it was not possible or required to obtain informed consent from the patients. The work was carried out in accordance with The Code of Ethics of the World Medical Association (Declaration of Helsinki).

Control samples (PIX CBC) used with the HemoScreen™ analyzer was obtained from R&D Systems (Minneapolis, MN, USA).

### Instrumentation

The Sysmex XN used in this study is a cell counter utilizing flow cytometry technology. The instrument uses sheet fluid to align the cells in a single file where which is exposed to a laser beam. The instrument collects information on total fluorescent/scattered light emitted by the cells. No subcellular data is attained. Values from the Sysmex XN for the study population are presented in Table 1.

**Table 1:** Basic values for the study population (57 females and 43 males)

	Median	(range)
Age (years)	59.8	(21–93)
RBC (10^12^/L)	4.59	(2.95–6.12)
HB (g/L)	137	(92–204)
HCT (fraction)	0.41	(0.28–0.6)
MCV (fL)	89.4	(69.8–112.4)
MCH (pg)	30	(21–40)
MCHC (g/L)	334	(304–357)
Platelets (10^9^/L)	256	(83–457)
WBC (10^9^/L)	6.7	(3.2–28)

The cell counts are presented as means and range and are from the Sysmex XN instrument

The HemoScreen utilizes a novel technology called viscoelastic focusing which aligns the cells in a single plane. The instrument internal optics acquire a large number of microscopic images of the focused cells. The pictures are then subjected to image analysis, differentiating the cell types and providing subcellular data to increase the specificity of the measurements.

### Statistical analysis

The coefficient of variation for the HemoScreen™ instrument and correlation between the methods was calculated with Excel 2016 (Microsoft, Seattle, WA, USA). Deming regression analysis was performed using Method Validator (Metz, France). Data are also presented as Bland-Altman plots [15].

## Results

### Coefficient of variation (CV) for the HemoScreen™ analyzer

Within day variation was calculated based on four measurements for each of the three control levels during a single day (Table 2).

**Table 2:** Within day coefficient of variation (CV) for the three controls

	Mean	CV (%)	Mean	CV (%)	Mean	CV (%)
RBC (10^12^/L)	2.7	1.5%	4.8	0.8%	5.5	0.5%
HB (g/L)	74	2.7%	160	1.1%	190	0.3%
HCT (fraction)	20.2	1.5%	40.4	0.9%	50.2	1.1%
MCV (fL)	73.7	0.5%	84.7	0.2%	91.2	0.9%
MCH (pg)	26.9	1.2%	33.5	0.5%	34.4	0.1%
MCHC (g/L)	365	1.4%	396	0.6%	378	0.9%

Each control was analyzed four times. The results are presented as mean and CV in percentage for white blood cells, red blood cells and platelets for each control

A total of 13 measurements analyzed daily for each of three controls were used to calculate the total CV (Table 3).

**Table 3:** Total coefficient of variation (CV) for the three controls. Each control was analyzed once daily for 14 days

	Mean	CV (%)	Mean	CV (%)	Mean	CV (%)
RBC (10^12^/L)	2.7	2.6	4.76	2.1	5.56	1.7
HB (g/L)	7.3	3.2	16.2	3.0	19.3	2.6
HCT (fraction)	19.9	3.4	40.4	2.2	50.6	2.0
MCV (fL)	73.7	0.9	84.8	0.6	91.0	0.7
MCH (pg)	27.2	1.7	33.9	1.4	34.7	1.3
MCHC (g/L)	36.8	1.8	40.0	1.3	38.1	1.4

The results are presented as mean and CV in percentage for each control

### Correlation between the two analyzers

The Deming correlation equation for WBC (10^12^/L) was WBC_HemoScreen™_ = 1.016* WBC_Sysmex_ + 0.34; r = 0.981. The 0.95 confidence interval (CI) for the slope was 0.986–1.047 and for the intercept 0.12–0.56. The mean difference between the two instruments was 0.44 × 10^12^/L (95% confidence interval 0.33–0.55).

**Fig. 1:**
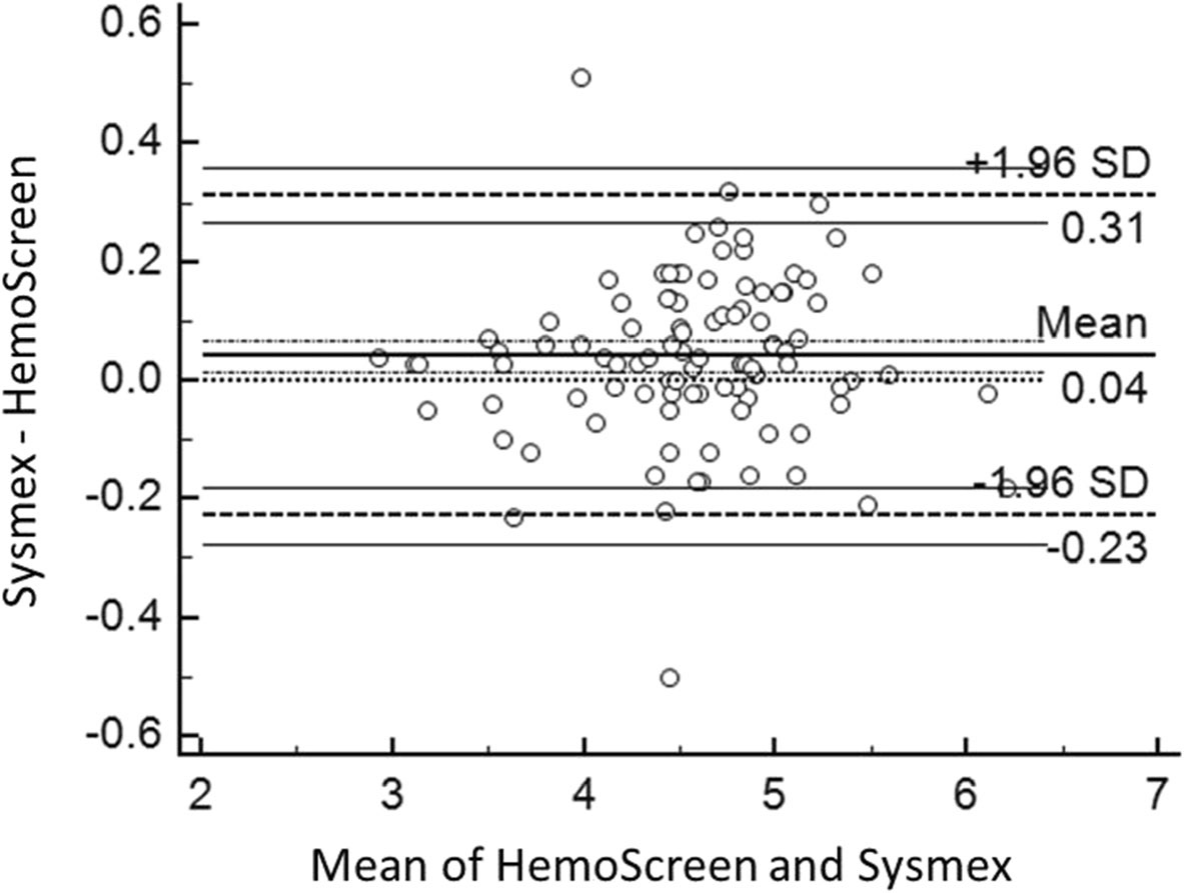
Bland-Altman plot for red blood cell counts (10^12^/L) with the mean of the two methods are plotted against the differences between the two methods. The horizontal lines show the mean difference between the two methods with 95% confidence intervals and limits of agreement with 95% confidence intervals. Mean bias between the methods was 0.04 × 10^12^/L

The Deming correlation equation for RBC (10^12^/L) was RBC_HemoScreen™_ = 0.988* RBC_Sysmex_ + 0.015; r = 0.974. The 0.95 confidence interval (CI) for the slope was 0.943–1.032 and for the intercept − 0.189 – 0.219. The mean difference between the two instruments was 0.04 × 10^12^/L (95% confidence interval − 0.23-0.31) (Fig. 1).

**Fig. 2:**
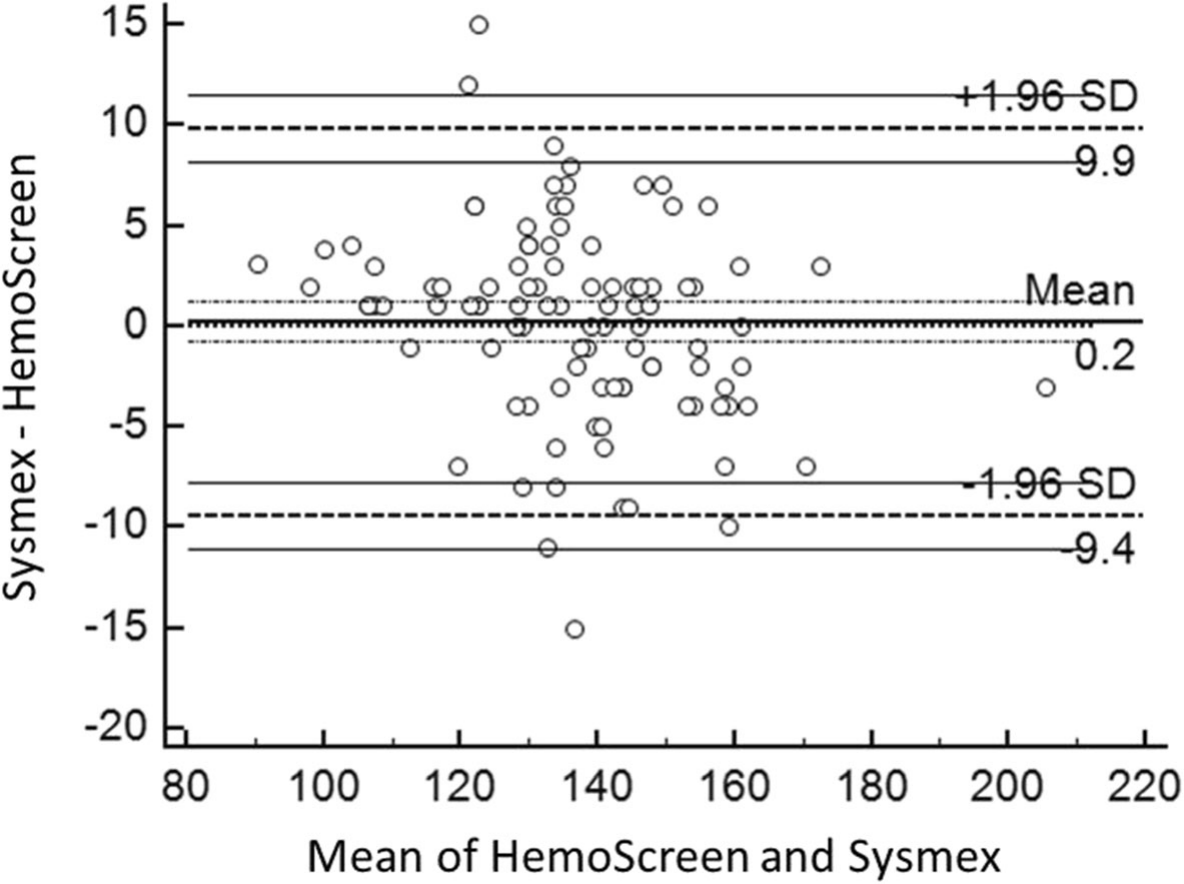
Bland-Altman plot for hemoglobin (g/L) with the mean of the two methods are plotted against the differences between the two methods. The horizontal lines show the mean difference between the two methods with 95% confidence intervals and limits of agreement with 95% confidence intervals. Mean bias between the methods was 0.2 g/L

The Deming correlation equation for hemoglobin (g/L) was Hemoglobin_HemoScreen™_ = 1.081* Hemoglobin_Sysmex_ − 11.25; r = 0.964. The 0.95 confidence interval (CI) for the slope was 1.037–1.125 and for the intercept − 17.31 – − 5.20. The mean difference between the two instruments was 0.2 g/L (95% confidence interval − 9.4-9.9) (Fig. 2).

The Deming correlation equation for hematocrite (fraction) was Hematocrite_HemoScreen™_ = 1.108* Hematocrite_ysmex_ − 0.0230; r = 0.958. The 0.95 confidence interval (CI) for the slope was 1.048–1.168 and for the intercept − 0.0473 – 0.0013. The mean difference between the two instruments was − 0.021.

**Fig. 3:**
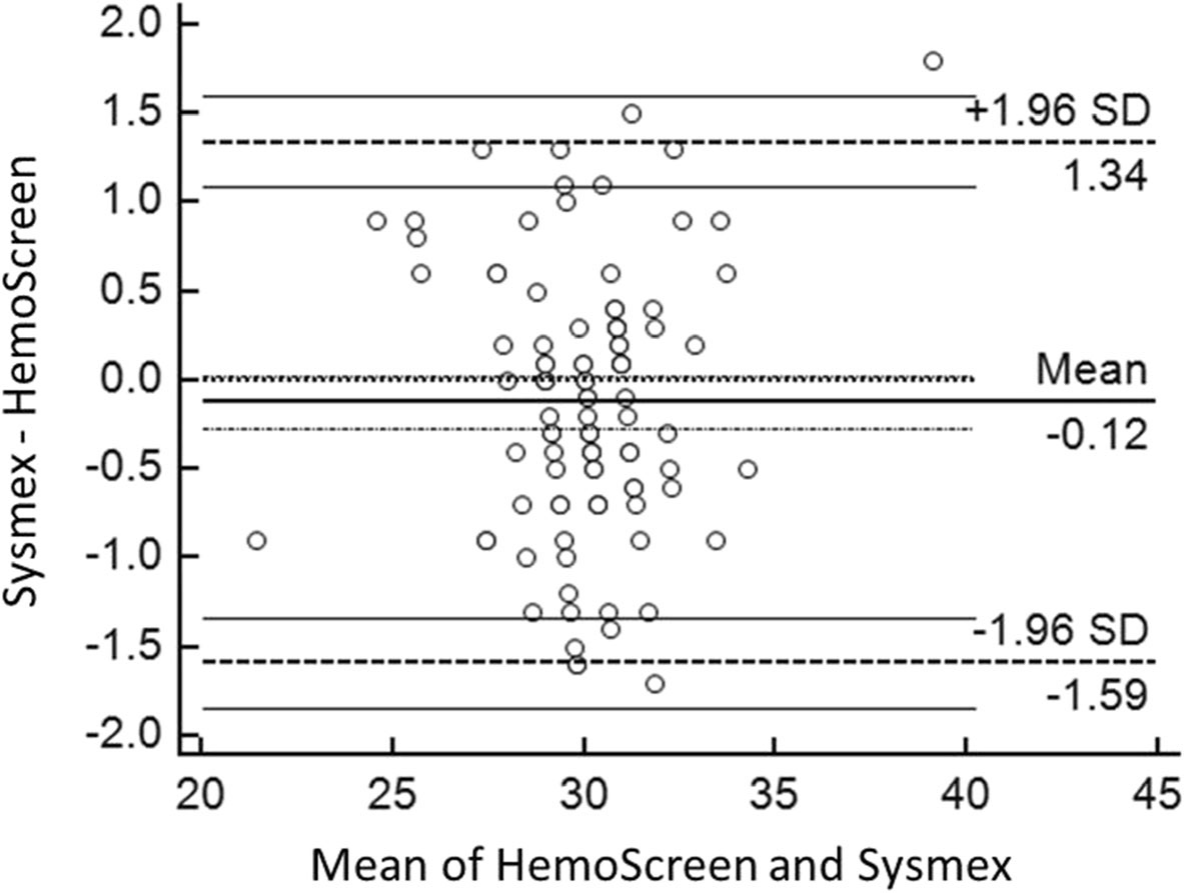
Bland-Altman plot for MCH (pg) with the mean of the two methods are plotted against the differences between the two methods. The horizontal lines show the mean difference between the two methods with 95% confidence intervals and limits of agreement with 95% confidence intervals. Mean bias between the methods was 0.12 pg

The Deming correlation equation for MCH (pg) was MCH_HemoScreen™_ = 0.978* MCH_Sysmex_ + 0.78; r = 0.939. The 0.95 confidence interval (CI) for the slope was 0.860–1.096 and for the intercept − 2.74 – 4.29. The mean difference between the two instruments was − 0.12 pg (95% confidence interval − 1.59-1.34) (Fig. 3).

The Deming correlation equation for MCHC (g/L) was MCHC_HemoScreen™_ = 0.901* MCHC_Sysmex_ + 15.9; r = 0.870. The 0.95 confidence interval (CI) for the slope was 0.809–0.994 and for the intercept − 14.9 – 46.7. The mean difference between the two instruments was 17.2 g/L.

**Fig. 4:**
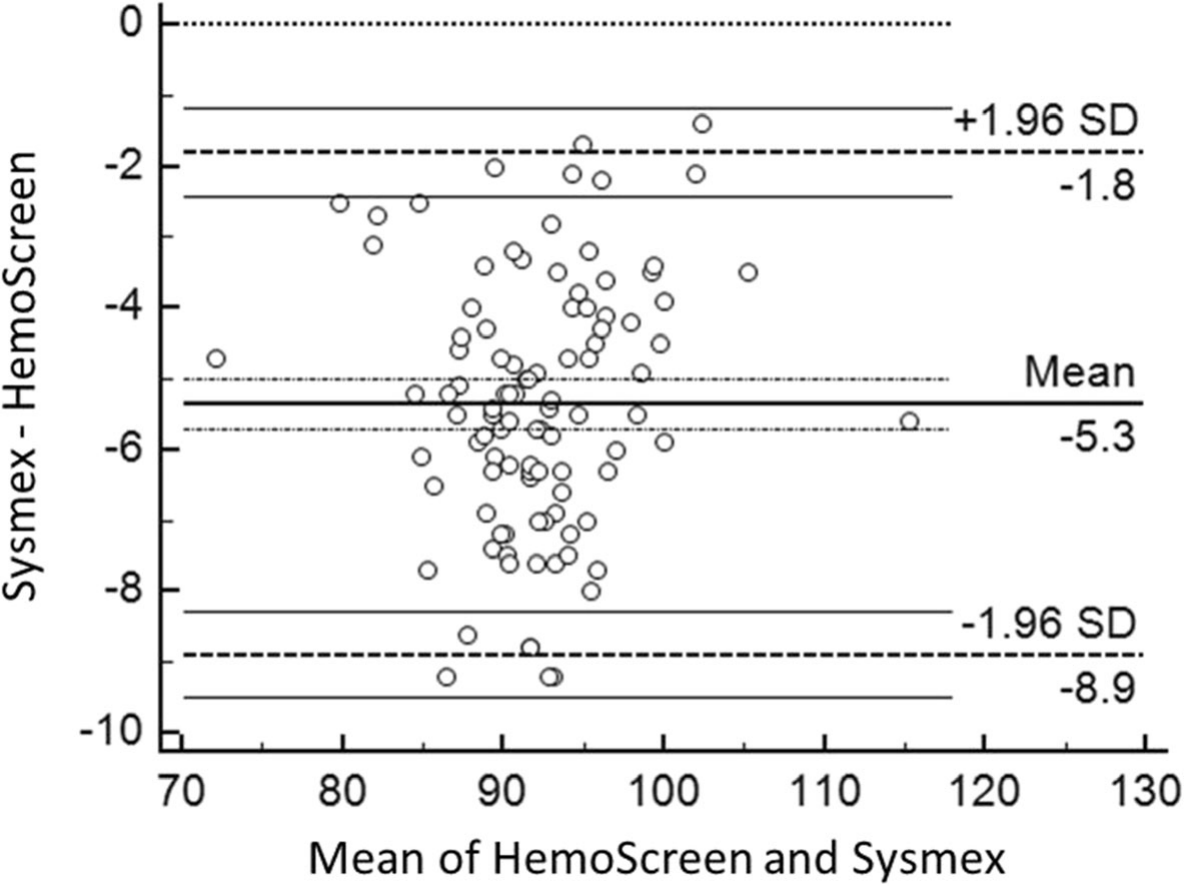
Bland-Altman plot for MCV (fL) with the mean of the two methods are plotted against the differences between the two methods. The horizontal lines show the mean difference between the two methods with 95% confidence intervals and limits of agreement with 95% confidence intervals. Mean bias between the methods was 5.3 fl

The Deming correlation equation for MCV (fL) was MCV_HemoScreen™_ = 0.963* MCV_Sysmex_ + 8.68; r = 0.946. The 0.95 confidence interval (CI) for the slope was 0.890–1.036 and for the intercept 2.12–15.24. The mean difference between the two instruments was − 5.3 fL (95% confidence interval − 8.9-(−)1.8) (Fig. 4).

The Deming correlation equation for platelet counts (10^9^/L) was Platelets_HemoScreen™_ = 0.964* Platelets_Sysmex_ + 25.7; r = 0.953. The 0.95 confidence interval (CI) for the slope was 0.890–1.038 and for the intercept 9.2–42.1. The mean difference between the two instruments was 17.2 × 10^9^/L.

The Bland-Altman plots of the comparisons between the two instruments for RBC, Hemoglobin, MCV and MCH are presented in Fig. 1-4.

## Discussion

**Fig. 5:**
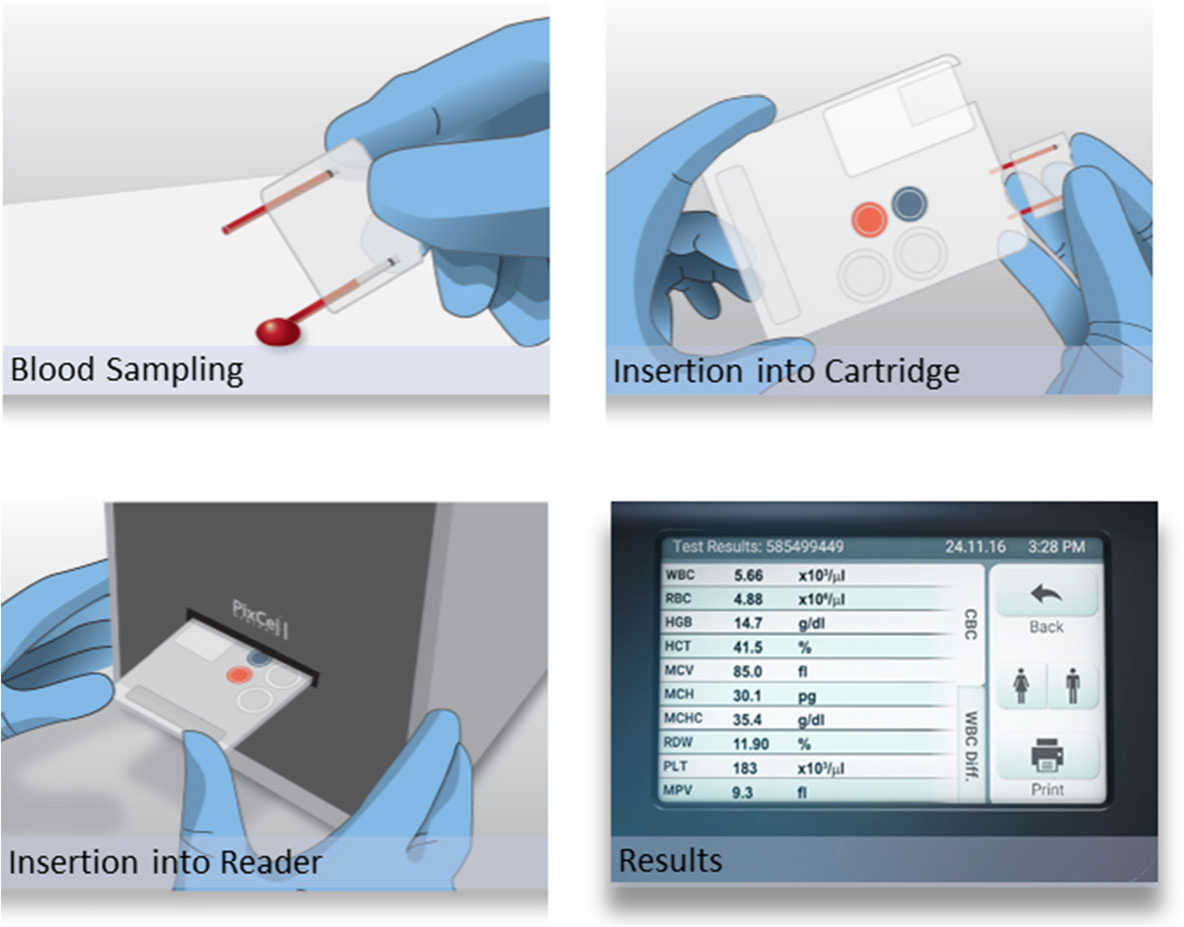
40 μl blood are collected using the HemoScreen capillary device (1), the capillaries are then introduced into the cartridge (2) and the cartridge is inserted into the HemoScreen instrument which started the analysis (3). After 6 min the results are displayed on the instrument screen (4). Image courtesy of PixCell Medical, Yokneam Ilit, Israel

In this study we compare two completely different techniques for analyzing blood cell counts. We compared the results from the cell counter Sysmex NX intended for large laboratories with the HemoScreen instrument which utilizes viscoelastic focusing and image analysis and is intended for point of care testing of blood cell counts. Due to the use of disposable cartridges, the HemoScreen instrument is very well adapted to point of care testing as it is easy to operate. The experience and skills required from the operator to run the system are similar to those needed to perform a home glucose test. This study presents a new technology for cell counting and differentiation. The same blood samples were used for both the measurements. The tubes were first analyzed on the Sysmex XN platform. After the primary analysis the tubes were collected from the Sysmex XN within 2 h for testing on the HemoScreen. The tubes were mixed on a Triomix mixer (Triolab, Mölndal, Sweden). 40 μL blood from the tubes were collected using the HemoScreen capillary device, the capillaries were introduced into the cartridge and the cartridge was inserted into the HemoScreen instrument which started the analysis (Fig. 5). We used venous tubes in the study to avoid the sample variation that may occur when using fingertip samples and to ensure that we got sufficient material for both measurements.

Point of care testing (POCT) can be used to gain rapid test results. By eliminating transport and laboratory processing times, POCT provides immediate access to test results, compared with delays of several hours that occur when the samples are sent to a central laboratory. These delays are longer than patients can be expected to wait in the GP’s office, and forces health care providers to spend time following up results with patients over the phone or during a subsequent visit, delaying treatment decisions. In Uppsala County the larger primary care centers are equipped with ABX micros cell counters (Horiba, Kyoto, Japan) mainly for rapid investigation of patients with suspected anemias. Cell counters are complex instrumentations, requiring careful maintenance. They work well in larger primary care centers equipped with laboratory technicians but are less well suited for the small primary care centers. The advantage of single use cartridges for the samples is that for instance a clot in the sample will only affect the cartridge and not the instrument. This new technology thus requires less maintenance and permit analysis of CBC parameters also at smaller primary care centers. The Swedish primary care depends heavily on POCT CRP testing for workup of patients with suspected infectious diseases and blood cell counts are therefore mainly used for patients with suspected anemia. We found a very good correlation between the RBC, hemoglobin, MCV and MCH results obtained with the HemoScreen™ and Sysmex NX instruments with r values between 0.939 and 0.974. We also observed good correlations for WBC and platelets.

There was a good correlation for MCV but a slightly larger bias than for RBC, hemoglobin and MCH. We observed a bias for MCV but it is not possible to decide which of the methods that provides the most correct MCV values. It is likely that the bias is due to differences in calibrations between the two instruments. It would be desirable that the instrument bias for MCV was reduced as alternate measurements on instruments with different calibrations can lead to diagnostic problems. Further studies are needed to investigate this bias and decrease it.

The Deming correlation (r = 0.870) was lower for MCHC than for the other studied parameters. In Swedish primary care this is less troublesome as less focus is on MCHC than the other parameters when investigating anemia.

There were also low intraday and total CV% values for the studied parameters. With an assay time for the HemoScreen™ instrument of approximately 6 min this would allow results during the initial consultation at the primary care center. During the study period we did not encounter any technical problems with the instrument and the instrument was easy to handle.

## Conclusion

The study indicates that the HemoScreen™ instrument can provide rapid and accurate analysis of blood cell parameters to shorten the workup time of anemia patients in primary care.

## Data Availability

The data sets used and/or analyzed during the current study are available from the corresponding author on request.

## Abbreviations

CI: Confidence interval
HCT: Hematocrit
HGB: Hemoglobin
MCH: Mean cell hemoglobin
MCHC: Mean cell hemoglobin concentration
MCV: Mean corpuscular volume
POCT: Point of care testing
RBC: Red blood cells
WBC: White blood cells
